# An Exploratory Study: Undergraduates’ Perspectives on how Threshold Concepts Influence Professional Identity

**DOI:** 10.1177/00084174231154747

**Published:** 2023-02-13

**Authors:** Shenae E. O’Mahony, Annette V. Joosten, Jennifer O’Brien

**Keywords:** Occupational therapy, Education, Professional identity, Professionalism, Occupation-based, Ergothérapie, fondé sur l’occupation, formation, identité professionnelle, professionnalisme

## Abstract

**Background.** Threshold concepts are key to professional identity development, transforming the way individuals think, act, and perceive the world. **Purpose.** To understand how occupational therapy students describe their professional identity, its importance, and how threshold concepts contribute to identity. **Method.** Mixed-method survey of final-year occupational therapy students (*n* = 58) at an Australian University. **Findings.** (i) High agreement on most identity and threshold questions; but up to 24% uncertain about confidence/competence in understanding specific concepts; (ii) occupation-focus is unique to our professional identity; (ii) identity develops over time; (iii) occupation-based, client-centered, and evidence-based practices are central to thinking like and becoming an occupational therapist; and (iv) practice education provides context for threshold concepts to be transformative. **Implications.** Identity is defined by a focus on occupation and its relationship to health. Traversing threshold concepts through academic and practice education is essential to developing professional identity.

## Résumé

**Description.** Les concepts seuils (*threshold concepts*) jouent un rôle central dans le développement de l’identité professionnelle, transformant la manière dont les personnes pensent, agissent et perçoivent le monde. **But.** Comprendre comment les étudiants en ergothérapie décrivent leur identité professionnelle, son importance, et la manière dont les concepts seuils y contribuent. **Méthodologie.** Enquête à méthode mixte auprès d’étudiants de dernière année en ergothérapie (*n* = 58) dans une université australienne. **Résultats.** (i) Un accord élevé sur la plupart des questions relatives à l’identité et aux seuils, mais jusqu’à 24 % d’incertitude quant à la confiance/compétence dans la compréhension de concepts particuliers; (ii) L’accent mis sur l’occupation est unique à notre identité professionnelle; (iii) L’identité se développe au fil du temps; (iv) Les pratiques fondées sur l’occupation, axées sur la clientèle et fondées sur des données probantes sont centrales pour penser comme un ergothérapeute et devenir un ergothérapeute; et (v) La formation à la pratique fournit un contexte pour que les concepts seuils soient transformateurs. **Conséquences.** L’identité est définie par l’accent sur l’occupation et sa relation avec la santé. Les concepts seuils qui traversent la formation universitaire et pratique sont essentiels pour le développement de l’identité professionnelle.

## Introduction

Professional identity refers to an individual's sense of self, informed by the values, attitudes, skills, and knowledge shared with others in their professional group (Adams et al., 2006; Caza & Creary, 2016). Identity development occurs over time and is influenced by practice education, role-modeling, professional socialization, academic learning, and assessment activities (Adams et al., 2006; Ashby et al., 2016; Boehm et al., 2015; Davis, 2006; Springfield et al., 2017; Toal-Sullivan, 2006; Whitcombe, 2013). Professional identity is significant, as it influences members’ thinking and behaviors and allows individuals to elucidate meaning and social contribution (Caza & Creary, 2016) and assert professional status (Clouston & Whitcombe, 2008; Turner & Knight, 2015; Whitcombe, 2013). Professional identity contributes to a sense of worth, self-efficacy, self-esteem, and pride (Caza & Creary, 2016), and safeguards against work-related stress and burnout (Edwards & Dirette, 2010). Occupational therapists report challenges in articulating identity (Fitzgerald, 2014; Kinn & Aas, 2009; Turner & Knight, 2015; Whitcombe, 2013) so it is important to understand undergraduate students’ professional identity development, and whether students can articulate their identity.

Occupational therapy practice is guided by occupational therapy conceptual models (Joosten, 2015) and an occupational perspective of health (Kinn & Aas, 2009; Whitcombe, 2013) that differs from dominant social and medical models of other health professions (Turner & Knight, 2015; Wilding & Whiteford, 2009). This difference can contribute to difficulty in describing their role, and in having others understand their professional identity (Ashby et al., 2016; Davis, 2006; Kinn & Aas, 2009). Occupational therapy students are in the formative stages of their professional identity development and, as reported by Nicola-Richmond et al. (2019) new graduates are still acquiring some threshold concepts.

Threshold concepts represent challenging notions that are central to a profession and transform students’ thinking, and ultimately how they perceive themselves and the world. Termed “threshold concepts” are “akin to a portal, opening up a new and previously inaccessible way of thinking” (Meyer & Land, 2003, p. 1) whereby the learner crosses a threshold to new understanding. These concepts can be troublesome to learn, and for some time, the learner exists within a liminal space, as they stand at the threshold oscillating between states of understanding (Fredholm et al., 2020; Land et al., 2014; Neve et al., 2016). Liminality is a transitional process characterized by the reformulation of meaning and reconstitution of identity, with existing views relinquished and replaced with a new alternative version of self (Land et al., 2014). Transformation is evident in knowing how to think and behave within your profession (Meyer & Land, 2003) and reflects cognitive and affective changes and new intellectual understanding (Rattray, 2016).

Debate exists about which concepts are transformative threshold knowledge for the profession (Fortune & Kennedy-Jones, 2014; Nicola-Richmond et al., 2016; Rodger & Turpin, 2011; Tanner, 2011). Client-centered practice; occupation-based practice; theory in practice; evidence-based practice; and critical thinking, reasoning, and reflecting are the concepts most reported in the occupational therapy literature, but not all are unique to occupational therapy. Less-reported concepts include occupational therapy role, discipline-specific skills/knowledge (Nicola-Richmond et al., 2016), and the development of professional identity (Tanner, 2011).

The links between professional identity and threshold concepts especially from the student perspective are not well reported in the literature. Previous studies have focused on student perspectives on threshold concepts (Nicola-Richmond et al., 2016), when threshold concepts are acquired (Nicola-Richmond et al., 2019), and the impact of role-emerging placements on threshold concepts and learning (Kaelin & Dancza, 2019), but not the link between threshold concepts and the development of professional identity. Therefore, the role threshold concepts may have in the education of occupational therapy students, who are in the formative stages of professional identity development, is of particular interest.

This study therefore addressed the following objectives:

1. to understand occupational therapy students' perceptions of professional identity, its role, and importance to practice; and
2. to explore how threshold concepts contribute to occupational therapy students’ professional identity.

## Method

A mixed-method survey incorporating quantitative and qualitative questions was used (Braun et al., 2021). This design was used as writing their responses to open-ended questions, in their own words enables participants to give rich descriptions of their experiences, and positioning (Braun et al., 2021), to provide rich explanations of the quantitative responses. The mixed-method online survey enabled the collection of a greater number of participant responses and perspectives.

Ethics approval was granted from the Australian Catholic University Human Research Ethics Committee (Ethics Register Number 2017-295E) in January 2018, with approval to survey students obtained from the Deputy Vice-Chancellor (Students, Learning and Teaching).

### Sampling and Recruitment

A convenience sample of Australian Catholic University students enrolled in at least one fourth-year unit in the undergraduate Bachelor of Occupational Therapy Program which operates nationally in Melbourne, North Sydney, and Brisbane as a single program: all campuses involved share the same prescribed texts, readings, and assignments; learning objectives; and tutorial and lecture content. In the first year, students completed foundational occupational therapy units and were introduced to social determinants and health-related content, and evidence-based practice with an occupation and participation focus. This was expanded in the following years with content across all occupational practice contexts, and across the lifespan, and was inclusive of indigenous, assistive technology, and recovery and health content. Teaching and assessment activities provided the students with opportunities to develop and apply their professional reasoning and reflective practices, apply occupational therapy conceptual models, interact with consumers, and participate in simulated and community and interprofessional engagement. Participating students had completed at least 700 of the World Federation of Occupational Therapists’ minimum required 1000 h of professional practice education in traditional and role-emerging settings.

### Data Collection

Data were collected via an online survey developed and disseminated using Qualtrics (2018) software. The survey was divided into three sections; (a) participant demographics, (b) professional identity, and (c) threshold concepts. Equal weighting was given to quantitative and qualitative survey data: the survey comprised closed questions addressing professional identity and the threshold concepts identified as transformative in the literature, with Likert scale responses (“strongly disagree,” “disagree,” “neither agree nor disagree,” “agree,” and “strongly agree”); and 15 open-questions (Supplement 1). To promote validity, questions were informed by the literature, and by the undergraduate curriculum of the university, drafted by the first author, reviewed by the second and third authors, and piloted with nine occupational therapy students from the 2017 cohort. Data collection occurred during February–March 2018, for a period of four weeks.

### Quantitative Analysis

Descriptive statistical analysis was conducted using SPSS (version 25; IBM Corporation, 2017). Measures of central tendency and dispersion were included to assist identification of patterns within the data set and to highlight areas of convergence and contention between participant responses.

### Qualitative Analysis

**Figure 1.**
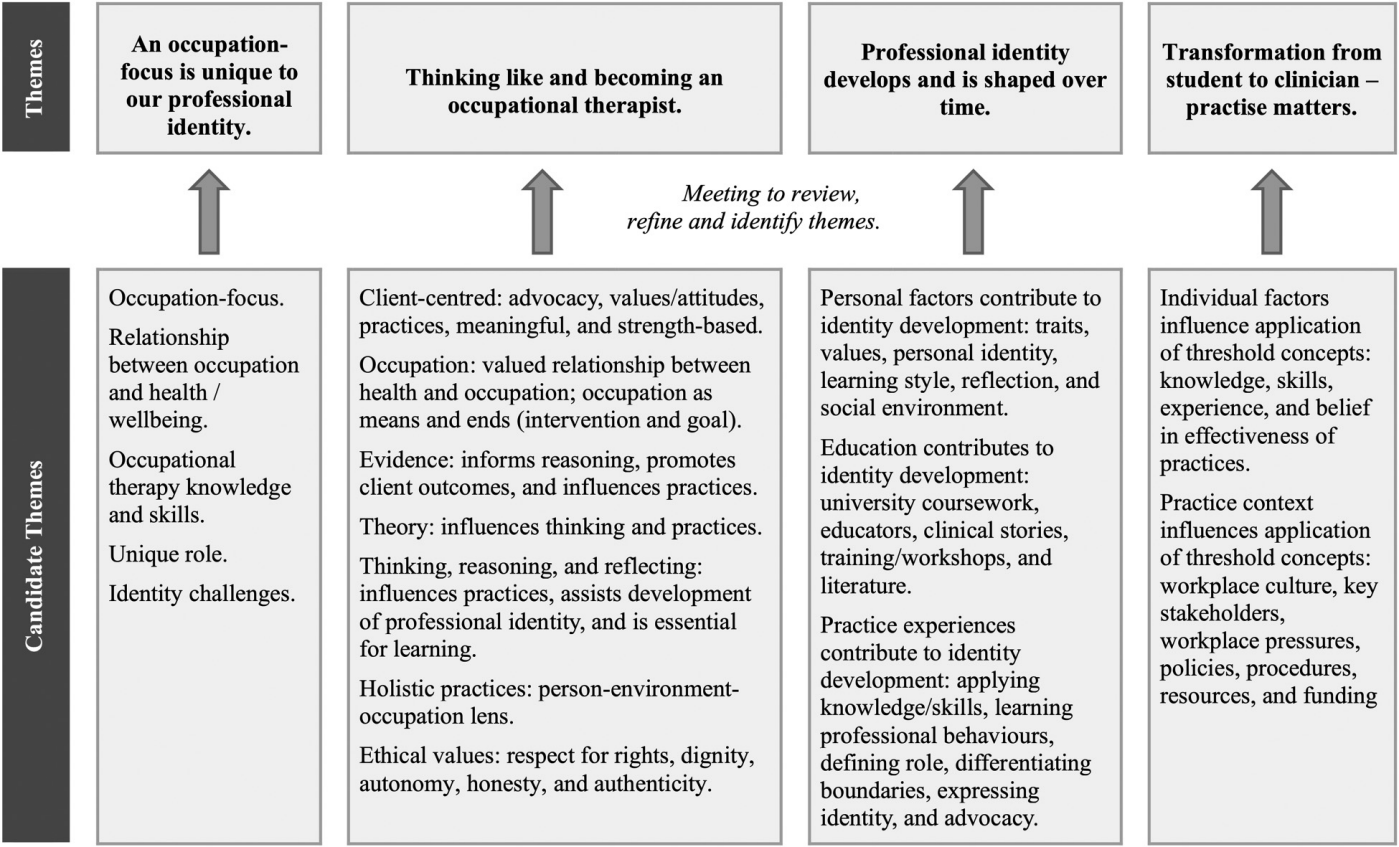
Refinement of qualitative themes.

Reflexive thematic analysis (RTA), guided by the procedures of Braun and Clarke (2006, 2013, 2019), was used to analyze text responses to open survey questions as it allows flexible application across a range of theoretical perspectives (Braun & Clarke, 2006). RTA can be used with a wide variety of data collection methods including open survey questions (Braun et al., 2021). The analysis required researchers’ immersion in the data using an iterative process and all researchers independently coded the data across the data set. The first author (researcher) was an honors student and first-year graduate as this study was written up, the second author has 35 years of experience as an occupational therapist in pediatric practice plus 15 years as an occupational therapy academic, and the third author has experience in mental health practice. The researchers then met to create a shared nuanced understanding of the data through discussion and the development of initial themes; and data that shared a main concept were developed into the final central organizing themes. The researchers’ experiences as occupational therapists provided the theoretical approach that guided the reflective, analytic process. An audit trail of the process was created to ensure credibility and dependability and Figure 1 presents the refinement of initial themes to create the final themes.

## Findings

### Participants

Of the 230 students eligible and invited to participate in the survey, 84 participants responded but 26 were excluded as only demographic data were provided resulting in 58 participants (response rate of 25%). Demographic data are presented in Table 1.

**Table 1:** Participant Demographics.

Variable		Frequency (*n*)	Percentage (%)
Age (years)	19–25	45	77.6
	26–30	7	12.1
	31–35	3	5.2
	36–40	1	1.7
	41–45	2	3.4
Sex	Male	3	5.2
	Female	55	94.8
Enrolment status	Part-time	7	12.1
	Full-time	51	87.9
Campus	Brisbane	12	20.7
	Melbourne	33	56.9
	North Sydney	13	22.4

### Quantitative Results Related to Professional Identity

The survey questions relating to understanding occupational therapy students’ perception of professional identity, role, and importance to practice, with the analysis of responses, are presented in Table 2. There was consensus (% represent agree and strongly agree responses) that professional identity influenced student thinking and behaviors (95%); was important for occupational therapy (93%); and ensured clear professional roles and disciplinary boundaries (79%). The majority of participants agreed that they had a clear understanding of their professional role (88%) and felt a sense of belonging in the profession (74%). Responses about whether students thought that occupational therapists had a well-defined professional identity varied (43% agreed, 28% disagreed, and 29% were unsure). Across all questions, there were very few strongly disagree/agree responses, but particularly in relation to occupational therapy theory in practice and thinking critically reasoning, and reflecting, 22% responded as neither agreeing/disagreeing on several questions. Detailed response rates and percentages are reported in Table 2.

**Table 2:** Likert Responses for Professional Identity.

Variable	Strongly disagree *n* (%)	Disagree *n* (%)	Neither agree nor disagree *n* (%)	Agree *n* (%)	Strongly agree *n* (%)	Median	IQR	*N*
Professional identity is important for occupational therapy practice.	1 (1.7)	0 (0.0)	3 (5.2)	21 (36.2)	33 (56.9)	5	1.0	58
Professional identity ensures clear professional roles and respect for disciplinary boundaries.	1 (1.7)	1 (1.7)	11 (19.0)	33 (56.9)	12 (20.7)	4	0.0	58
Identity influences the way I think and behave in the profession.	1 (1.7)	0 (0.0)	2 (3.4)	35 (60.3)	20 (34.5)	4	1.0	58
Occupational therapists have a well-defined and clearly understood professional identity.	2 (3.4)	14 (24.1)	17 (29.3)	22 (37.9)	3 (5.2)	3	2.0	58
I have a clear understanding of the role of occupational therapy.	0 (0.0)	1 (1.7)	6 (10.3)	37 (63.8)	14 (24.1)	4	0.0	58
I feel confident explaining my role to clients/families.	0 (0.0)	2 (3.4)	8 (13.8)	36 (62.1)	12 (20.7)	4	0.0	58
I am confident in explaining the role of occupational therapy to other professionals.	1 (1.7)	3 (5.2)	13 (22.4)	33 (56.9)	8 (13.8)	4	1.0	58
I feel a sense of belonging within the occupational therapy profession.	1 (1.7)	1 (1.7)	13 (22.4)	28 (48.3)	15 (25.9)	4	1.5	58

*Note*. IQR = interquartile range.

### Quantitative Results Related to Threshold Concepts

The survey questions relating to understanding occupational therapy students’ perception of how threshold concepts contribute to occupational therapy professional identity and the responses are reported (% represent agree and strongly agree responses) and presented in detail in Table 3. Professional identity includes the values, knowledge, skills, and attitudes of a profession. Participants were asked about the value they assigned to each of the threshold concepts and their knowledge and skill in relation to these concepts. The threshold concepts were highly valued by participants; they considered client-centered (79%–98% agreement on the four questions), occupation-based (79%–98% agreement on the three questions), and evidence-based practices (83%–96% agreement on three questions; but only 67% agreed they felt comfortable asking supervisors about their evidence-based practices). Theory, thinking, and self-reflection were also important to practice; ranging from agreeing they could explain clinical decisions (71%) to reflection being important to practice (91%). Participants generally agreed they were confident in their knowledge and skills pertaining to client-centered, occupation-based, and evidence-based practices, occupational therapy theory, reflection, and articulating clinical reasoning to colleagues and clients. Participants were asked to rank the concepts in order of importance; they assigned greater weightings to client-centered, occupation-based, and evidence-based practices compared with critical thinking, reasoning, reflecting, and occupational therapy theory.

**Table 3:** Likert Responses for Threshold Concepts.

Variable	Strongly disagree *n* (%)	Disagree *n* (%)	Neither agree nor disagree *n* (%)	Agree *n* (%)	Strongly agree *n* (%)	Median	IQR	*N*
**Threshold concept 1: Occupation-based practice**								
Maintaining an occupation focus is important to me on placement.	0 (0.0)	0 (0.0)	1 (1.7)	27 (46.6)	29 (50.0)	5	1.0	57
University content has provided a rich understanding of occupation and its relationship with health.	0 (0.0)	0 (0.0)	0 (0.0)	29 (50.0)	28 (48.3)	5	1.0	57
I feel confident explaining the value and meaning of occupation to colleagues, clients, and families.	0 (0.0)	0 (0.0)	7 (12.1)	29 (50.0)	21 (36.2)	4	1.0	57
My colleagues (other professionals) on placement are supportive of the occupation-based practice.	0 (0.0)	4 (6.9)	10 (17.2)	34 (58.6)	9 (15.5)	4	0.0	57
**Threshold concept 2: Client-centered practice**								
I understand what being client-centered involves and how to implement this.	0 (0.0)	0 (0.0)	1 (1.7)	37 (63.8)	19 (32.8)	4	1.0	57
Listening to and prioritizing clients' values are important to my practice.	0 (0.0)	0 (0.0)	0 (0.0)	15 (25.9)	42 (72.4)	5	1.0	57
I understand the therapeutic use of self and how to apply it in practice.	0 (0.0)	1 (1.7)	10 (17.2)	31 (53.4)	15 (25.9)	4	1.0	57
**Threshold concept 3: Occupational therapy theory and practice**								
Occupational therapy theories are integral to my practice.	0 (0.0)	0 (0.0)	7 (12.1)	36 (62.1)	14 (24.1)	4	0.0	57
I feel confident applying theories and frames of reference in practice.	0 (0.0)	5 (8.6)	14 (24.1)	26 (44.8)	12 (20.7)	4	1.0	57
I use occupational theories to justify clinical decisions.	0 (0.0)	3 (5.2)	11 (19.0)	29 (50.0)	14 (24.1)	4	0.0	57
I am likely to use conceptual models on placement.	2 (3.4)	3 (5.2)	13 (22.4)	28 (48.3)	11 (19.0)	4	1.0	57
**Threshold concept 4: Evidence-based practice**								
I value evidence-based practice.	0 (0.0)	0 (0.0)	1 (1.7)	24 (41.4)	32 (55.2)	5	1.0	57
I have the necessary knowledge to undertake a review of the literature.	1 (1.7)	1 (1.7)	5 (8.6)	33 (56.9)	17 (29.3)	4	1.0	57
I am likely to implement evidence-based practices on placement.	0 (0.0)	0 (0.0)	9 (15.5)	32 (55.2)	16 (27.6)	4	1.0	57
I am comfortable asking my supervisor/colleagues about the evidence supporting their practices.	0 (0.0)	7 (12.1)	11 (19.0)	27 (46.6)	12 (20.7)	4	1.0	57
**Threshold concepts 5: Thinking critically, reasoning, and reflecting**								
Reflection is important to my practice.	0 (0.0)	1 (1.7)	1 (1.7)	28 (48.3)	25 (43.1)	4	1.0	55
I am knowledgeable about specific reflective practices or models which may be used to guide reflection.	0 (0.0)	1 (1.7)	7 (12.1)	29 (50.0)	18 (31.0)	4	1.0	55
I can articulate my clinical reasoning to my supervisor and other professionals on placement.	0 (0.0)	0 (0.0)	12 (20.7)	36 (62.1)	7 (12.1)	4	0.0	55
I can justify and explain clinical decisions to clients.	0 (0.0)	1 (1.7)	13 (22.4)	32 (55.2)	9 (15.5)	4	0.5	55
My colleagues and/or placement supervisors support critical thinking and reflective practices.	1 (1.7)	0 (0.0)	4 (6.9)	28 (48.3)	22 (37.9)	4	1.0	55

*Note*. IQR = interquartile range.

### Qualitative Results

Four major themes were developed: Themes 1 and 2 relate to professional identity: (a) an occupation-focus is unique to our professional identity, and (b) professional identity develops and is shaped over time. Themes 3 and 4 explored the link between professional identity and threshold concepts: (c) thinking like and becoming an occupational therapist, and (d) transformation from student to clinician—practice matters.

#### Theme 1: An Occupation-Focus is Unique to our Professional Identity

Before being presented with the quantitative survey content, participants were asked to describe occupational therapy professional identity. Occupation, occupational role, participation enabling engagement, meaningful occupation, and “occupation, health, and well-being” were terms most frequently used to describe what it was to be an occupational therapist. Attitudes, skills, and knowledge were considered to be “unlike any other health-role” (P8). The role of the occupational therapist was to improve occupational “functioning, participation and overall quality of life” (P13) and to increase “the independence of people who are affected by illness, disability or disease” (P16). This was also reported as enabling people “to engage in meaningful occupations” (P9) and/or “roles that are important to them” (P57) and to provide “care that is determined by the clients wants, needs and desires” (P3). As P5 reported, central to this role, was the “belief that every person has the right to participate in meaningful occupations.”

Challenges in describing the occupational therapy role were also related to our occupational focus:

an undervaluing of meaningful participation over medical health within the health field [*sic*] and general population, confusion about our abilities and roles within and outside the occupational therapy discipline, and a lack of knowledge about occupational therapy within the general population. (P13)

However, the breadth and diversity of practice settings and the added challenge of being a relatively new profession were also perceived as making it difficult for participants to describe their role, or have it understood, by others external to the profession. P40 reported that “it is still not a well-known career and our roles and identities aren't very clear to the community”; and P25 discussed misconceptions or “common assumptions that we are ‘like physios’ or help people find jobs.”

#### Theme 2: Professional Identity Develops and is Shaped Over Time

Personal factors, university education, and practice education contribute to professional identity development.

##### Personal Factors

Participants reported that professional development was unique to the individual and that identity development was influenced by personality traits, values, personal identity, learning style, and reflective practices. “We require a certain type of personality that is empathetic, engaging and patient” (P40); and “one must be selfless, kind and humble” (P34). Ethical values such as respect for human rights, dignity, autonomy, honesty, and authenticity, were considered both personal and professional values. Identity was influenced by the individual's unique social environment including family, friends, peers, academic and practice educators, occupational therapists, and other professionals. Summarized by Participant 51, professional identity was a “fluid concept” and develops from “personal values and how they interact with others and the values of my future profession.” Personal experiences including employment, community engagement, social interactions, life events, and transitions were also considered influential.

##### University Education

Professional identity development was reportedly influenced by educational experiences, including university coursework, training/workshops, and through occupational therapy literature, which scaffolded student understanding of the threshold concepts: “I have learnt valuable knowledge about the profession from lectures, tutorials, placement and by other students sharing their experiences” (P33). University education was considered particularly influential when educators shared their stories, relating concepts to clinical experiences and discussing reasoning and values: “through their clinical reflections I have gained a better understanding of how important my professional identity is” (P55), and “I have looked to them to see what kind of practitioner I want to be” (P30).

##### Practice Education

University education provided theoretical knowledge, practical skill development, and opportunities to develop professional reasoning but professional practice education was characterized as “essential” (P5 and 12), “invaluable” (P17), and “integral to developing professional identity” (P39). Authentic, real-life opportunities to practice, develop, and apply knowledge, skills, and professional behaviors in context, were thought essential to traversing conceptual, transformative thresholds. Participant 3 explained:

Professional practice placements allow students to translate their knowledge into practice, and in doing so are able to learn about the unique role that OT can play across many different settings. Placements also allows students to understand the role of OT in a multidisciplinary context and may better understand how OT differs to other roles. By explaining the role of OT to clients, students are able to better grasp this definition. (P3).

Engagement in communities of practice allowed students to “get a feel for the way those within and outside your profession react, describe, and discuss occupational therapy” (P13); and that working alongside other communities of practice helped to “differentiate between other professions” (P39). Furthermore, practice education challenged participants to express their identity, or as Participant 41 stated, “to articulate my understanding … and my role as an OT in a number of contexts.” P54 reflected, “I did not fully realise how important the professional identity and promotion of the role of the OT was until I was in a practice placement setting.” This sentiment was echoed by Participant 15 who stated that by “interacting with individuals who may have never experienced an OT, or who may have a limited understanding of the role of an OT, we are provided with opportunities to grow our confidence in portraying our profession and our identity.”

#### Theme 3: Thinking Like and Becoming an Occupational Therapist

Participants reported occupation-based, client-centered, and evidence-based practices as central to professional identity development.

##### Occupation-Based Practice

Participants reported that in understanding the importance of occupational roles and recognizing occupation-based practices as inherently client-centered, they understood and valued the influence of occupation on health, well-being, and quality of life, and that they were thinking like an occupational therapist. “Our ethos is that we have a right to engage in meaningful occupations…, and well-being correlates with participating in occupation” (P45). Participants emphasized the importance of a holistic approach to assessment and intervention and recognized occupational participation as both an intervention and outcome of therapy. Participants described the holistic practice as evaluating “the fit between the person, environment and occupations” (P27): “to see the whole person in consideration to their ever-changing environments, their roles, routines, identity, and spirituality” (P5) and that this was unique to occupational therapy identity. Central to occupation-based practice was the idea that people were unique occupational beings and that understanding the interrelationship between person, environment, and occupation afforded a unique lens for occupational therapy.

##### Client-Centered Practice

Although not unique to occupational therapy, being client-centered was reported as being core to occupation-based practice. Participants reported that being client-centered meant valuing the uniqueness of the person, their preferences, and respect for human dignity, and this was central to how they viewed their profession. Participants identified occupational therapy as tailored to the individual and/or family, and emphasized the importance of relationships, collaboration, and validation of strengths: “Client-centred practice involves developing a relationship …, targeting therapy or interventions to address their goals” (P51), engaging the client as “an active participant in planning their recovery” (P40), and the need to “empower and advocate for the client … to capitalise on their strengths” (P3).

##### Evidence-Based Practice

The participants reported that as their professional identity developed, they understood and valued how evidence informed their professional reasoning, promoted occupation-focused client outcomes, and ensured “interventions provided are effective, safe and current” (P37). Participants 4 and 30 identified themselves as being an “evidence-based practitioner.” Participants also reported that occupational therapy literature informed professional identity and influenced the application of other threshold concepts in practice.

Participants did not regard “theory in practice,” and “critical thinking, reasoning, and reflecting” as threshold concepts or as concepts that helped them describe their role but reported them as skills that enabled the key threshold concepts to be traversed, and as such were influential to the development of identity.

##### Theory in Practice

Participants described the theory and the application of occupational therapy conceptual models as providing them with knowledge and a changed understanding of what it was to be, and to reason as, an occupational therapist: “it [theory] has become an ingrained way of thinking due to constant use within course work” (P15), and “we always use theory implicitly or by second nature” (P2). Participants 48 and 12, commented that theory “shaped” or altered their thinking and approaches to practice; and theories were thought to “underline most of the decisions made in practice” (P19).

##### Critical Thinking, Professional Reasoning, and Reflecting

Rather than being descriptors of identity, participants recognized critical thinking, professional reasoning, and reflecting as underlying transformative skills required for other threshold concepts to be mastered, and for the formation of professional identity. These skills were valued for their contribution to “the learning process” (P39) and ongoing development: “Critical thinking, reasoning and reflecting are vital for improving as an OT” (P15), and reflection “helps professional identity to develop” (P24).

#### Theme 4: Transformation from Student to Clinician—Practice Matters

Participants reported that professional identity developed in the university setting, but that practice education provided the opportunity for threshold concepts to be transformative. Participant 52 described practice education as

very important. I know for me it wasn't until I was able to put the theory that we had learn't [sic] into practice that I truly understood how incredible our role can truly be in a range of different environments.

Participants were motivated to apply their new understandings in context but recognized that they faced challenges and needed support to do so. Examples they provided were needing support to feel confident to negotiate and collaborate with clients when there were potential conflicts between being client-centered and evidence-based, as described by Participant 3: “recommending something that is beneficial for the client but they are hesitant to comply.” Similarly, “if the client goals are harmful or if the client does not think of any goals” (P33). Participants recognized the need to practice and observe how to negotiate when disagreement existed between stakeholders (clients, families, and health professionals), as they knew their role required the “ability to work with all stakeholders involved to advocate for what the client wants and needs” (P41).

At the organizational level, workplace culture was considered highly influential to student opportunity and confidence, in applying new learning. Students recognized that the organization and professionals had preferred practices, attitudes, values, and knowledge, which at times differed from their own, and that this challenged their thinking, values, and application of practices. Experiences varied, some participants felt “supported by staff” (P33) for specific practices, and others reported challenges associated with “set interventions” (P3) or “not using the most up to date interventions” (P4); supervisors who “do not seem to know what frame of reference they are working within, or seek to understand what is best practice in their area” (P5); and reluctance “to change their ways” (P40). Furthermore, perceived differences in “status as a student” (P17), influenced confidence in challenging workplace culture and existing practices: “It can be hard to challenge more senior OTs on the evidence behind their practice” (P47). Additionally, workplace pressures presented challenges including policies/procedures, limited resources, time restrictions, caseload demands, and pressure to discharge clients. Participants valued supervisors who provided clear expectations, supervised reflection, and timely and constructive feedback in helping them learn the role of an occupational therapist. Moreover, the supervisor's values, attitudes, knowledge, and experience influenced the practices they modeled. For example, participants reported that “it is a lot easier to discuss occupation-based practice, theory, etc.” (P5) when the supervisor advocates for these practices; without this support, students found it “difficult to be confident in using the approach” (P54).

## Discussion

The quantitative and qualitative findings from this exploratory study suggest that acquiring threshold concepts is linked to professional identity. However, unlike recently graduated and experienced therapists, final-year occupational therapy students are not competent in all threshold concepts, confirming earlier findings (Nicola-Richmond et al., 2019). In the current study, most participants agreed that being occupation-based was unique to occupational therapy identity and there was high agreement that client-centered, occupation-based practice and evidence-based practices were the concepts identified as being transformative. In contrast, many students neither agreed nor disagreed feeling confident or competent in areas of clinical reasoning and reflective and critical thinking, and applying theories and frames of reference in practice. The students reported these more troublesome concepts as knowledge and skills foundational and necessary for the key threshold concepts to be understood and transformative, but not as threshold concepts. This finding raises the need for fourth-year curriculum content, including professional practice experiences, that support the students to traverse these concepts to be work ready, under supervision and mentorship, on graduation.

The findings also confirmed earlier studies that reported client-centered and evidence-based practices as threshold concepts central to occupational therapy professional identity, but that they are not unique to occupational therapy (Kinn & Aas, 2009; Takashima & Saeki, 2013). Occupational therapy identity was distinguished from other professions, by its focus on occupation and its relationship to health. An indication that the students traversed this threshold concept was provided in their descriptions of thinking like occupational therapists, in their understanding of person, environment, and occupation as core to an occupational perspective on health (Njelesani et al., 2014), and that occupational therapists understand individuals as occupational beings engaged within unique contexts (Joosten, 2015).

Students perceived that identity developed from the interaction of personal factors, academic curricula, and practice experiences, which enable threshold concepts to be traversed, and is consistent with previous studies (Boehm et al., 2015; Davis, 2006; Springfield et al., 2017; Toal-Sullivan, 2006; Whitcombe, 2013).

Confirming the findings of previous studies (Adams et al., 2006; Boehm et al., 2015; Toal-Sullivan, 2006), participants reported that professional identity developed through authentic learning experiences. Professional practice opportunities enabled them to practice, explain their role, and learn from others. Students reported that these opportunities helped them understand the threshold concepts and start to think and see themselves as occupational therapists. Professional practice was regarded as particularly important to developing professional reasoning when they experienced incongruence between threshold concepts, such as being client-centered and evidence-based when the client wanted something that was not best practice. Practice education enabled them to see how this was managed in situ and explained to the client. This is of interest and in contrast to recent evidence about role-emerging placements and the benefits of students having to find their own way of doing things and developing their own reasoning and reflection, and that although uncomfortable learning for some, it may be transformative learning (Kaelin & Dancza, 2019). Further research/comparison of experiences in both traditional and role-emerging/long-arm supervision contexts is warranted, to improve understanding of the contribution both models of practice education have in student's identity development.

The findings of the current study suggest the need for academic and practice educators’ collaboration in facilitating students’ mastery of threshold concepts and supporting transformation from student to occupational therapist. Practice educators have been reported to influence the formation of students’ values and attitudes toward theoretical knowledge through their own use of theoretical language (Towns & Ashby, 2014). More overt use of occupational therapy conceptual models and theories provides a structure for occupational therapists, subsequently strengthening the articulation of the profession's distinct occupational perspective (Joosten, 2015; Njelesani et al., 2014). Time constraints, resources, and context demands all impact supervision practices, but strong collaboration between all educators is required to cocreate or determine ways to support students, to facilitate students’ mastery of threshold concepts and identity transformation.

### Limitations

The questionnaire variant of the mixed methods design and convenience sampling promoted project feasibility; but with a lower-than-anticipated response rate limiting transferability to other universities. However, a thick description of context and participants; and using a survey informed by the literature, piloted, and reviewed; provided a preliminary understanding of student perception that warrants further research. Caution is needed in interpreting the results as many eligible students did not participate and those more comfortable with the threshold concepts may have participated. This study only recruited participants from one university and did not explore the perspectives of graduate-entry master's students.

## Conclusion

Findings emphasize that threshold concepts are difficult to understand and not fully understood by all final-year students. This is because they are complex, troublesome, and can be difficult to understand. Participants identified the importance of practice education helping them understand the concepts and their identity development. The use of a shared theoretical language by educators and overt use of occupational therapy conceptual models was reported by the students as supporting their understanding of theoretical concepts and professional identity development. Including other universities and professional practice providers in further research to explore practices is warranted.

## Key Messages

- Threshold concepts are integral to occupational therapy students' descriptions of professional identity and contribute to its development.
- Practice education provides context for threshold concepts to be traversed and further research is needed to understand the curriculum content that ensures work-ready graduates.
- An occupational perspective of health which considers the interrelationship of person, environment, and occupation affords a unique identity and language for the profession.

## Supplemental Material

sj-docx-1-cjo-10.1177_00084174231154747 - Supplemental material for An Exploratory Study: Undergraduates’ Perspectives on how Threshold Concepts Influence Professional Identity Click here for additional data file. Supplemental material, sj-docx-1-cjo-10.1177_00084174231154747 for An Exploratory Study: Undergraduates’ Perspectives on how Threshold Concepts Influence Professional Identity by Shenae E. O’Mahony, Annette V. Joosten and Jennifer O’Brien in Canadian Journal of Occupational Therapy
