# Supplementary material for: An Exploratory Study: Undergraduates’ Perspectives on how Threshold Concepts Influence Professional Identity
Source: Can J Occup Ther. 2023 Feb 13;90(4):374–83. doi: 10.1177/00084174231154747 (PMC10647904; doi:10.1177/00084174231154747)
Supplement: sj-docx-1-cjo-10.1177_00084174231154747 - Supplemental material for An Exploratory Study: Undergraduates’ Perspectives on how Threshold Concepts Influence Professional Identity [file sj-docx-1-cjo-10.1177_00084174231154747.docx]

Qualitative Survey Questions

**Professional Identity**

*Professional identity refers to the way you perceive your work role. It includes the values, attitudes, knowledge, skills and understanding of work role that you share with other people in your profession.*

- Describe your professional identity as an occupational therapy student.
- What do you think are the most important values in the occupational therapy profession?
- In which units of your course do you think professional identity has been taught?
- How important do you think professional practice placements are to professional identity development?
- What else has influenced your professional identity development?

**Threshold Concepts**

- Define occupation-based practice.
- What factors influence your confidence using an occupation-based approach?
- Define client-centred practice.
- What factors influence your confidence providing client-centred practice?
- What factors influence your use of theory in practice?
- What factors influence your use of evidence-based practices?
- What factors influence your critical thinking and clinical reasoning?
- What factors influence your reflection on practice?
- Please rank each threshold concept in order of importance and comment on why you ranked the concepts in this order. Consider the concepts that you ranked highest and lowest, why are these concepts more, or less important to you?
- Is there anything else that you think effects your ability to confidently apply these concepts in practice?
